# Glucosamine-Modified Mesoporous Silica-Coated Magnetic Nanoparticles: A “Raisin-Cake”-like Structure as an Efficient Theranostic Platform for Targeted Methotrexate Delivery

**DOI:** 10.3390/pharmaceutics15102491

**Published:** 2023-10-19

**Authors:** Fatemeh Farjadian, Zahra Faghih, Maryam Fakhimi, Pooya Iranpour, Soliman Mohammadi-Samani, Mohammad Doroudian

**Affiliations:** 1 Pharmaceutical Sciences Research Canter, School of Pharmacy, Shiraz University of Medical Sciences, Shiraz 71468-64685, Iran; smsamani@sums.ac.ir; 2 Shiraz Institute for Cancer Research, School of Medicine, Shiraz University of Medical Sciences, Shiraz 71348-45550, Iran; faghihz@sums.ac.ir (Z.F.); maryamfakhimi67@gmail.com (M.F.); 3 Medical Imaging Research Center, Department of Radiology, Shiraz University of Medical Sciences, Shiraz 71936-13311, Iran; pooya.iranpour@gmail.com; 4 Department of Pharmaceutics, School of Pharmacy, Shiraz University of Medical Sciences, Shiraz 71468-64685, Iran; 5 Department of Cell and Molecular Sciences, Faculty of Biological Sciences, Kharazmi University, Tehran 15719-14911, Iran

**Keywords:** magnetic nanoparticle, mesoporous silica, anticancer, methotrexate, MRI, theranostic

## Abstract

This study presents the synthesis of glucosamine-modified mesoporous silica-coated magnetic nanoparticles (MNPs) as a therapeutic platform for the delivery of an anticancer drug, methotrexate (MTX). The MNPs were coated with mesoporous silica in a templated sol–gel process to form MNP@MSN, and then chloropropyl groups were added to the structure in a post-modification reaction. Glucosamine was then reacted with the chloro-modified structure, and methotrexate was conjugated to the hydroxyl group of the glucose. The prepared structure was characterized using techniques such as Fourier transform infrared (FT-IR) spectroscopy, elemental analysis (CHN), field emission scanning electron microscopy (FESEM), transmission electron microscopy (TEM), dynamic light scattering (DLS), a vibrating sample magnetometer (VSM), and X-ray diffraction (XRD). Good formation of nano-sized MNPs and MNP@MSN was observed via particle size monitoring. The modified glucosamine structure showed a controlled release profile of methotrexate in simulated tumor fluid. In vitro evaluation using the 4T1 breast cancer cell line showed the cytotoxicity, apoptosis, and cell cycle effects of methotrexate. The MTT assay showed comparable toxicity between MTX-loaded nanoparticles and free MTX. The structure could act as a glucose transporter-targeting agent and showed increased uptake in cancer cells. An in vivo breast cancer model was established in BALB/C mice, and the distribution of MTX-conjugated MNP@MSN particles was visualized using MRI. The MTX-conjugated particles showed significant anti-tumor potential together with MRI contrast enhancement.

## 1. Introduction

Nanomedicine is an emerging drug delivery technology that has revolutionized the traditional treatment of serious diseases [1,2,3,4]. Within this technology, considerable attention has been paid to the development of targeted nanoformulations for cancer treatment, with the aim of minimizing adverse effects on healthy organs [5,6]. Customized nanoparticles of different origins have been recruited for the delivery of anticancer agents by passive or active targeting [7,8]. Theranostics is an innovative concept that aims to combine diagnostics and therapeutics in a single platform [9,10]. Magnetic resonance imaging (MRI) is a non-invasive diagnostic tool commonly used to detect some important clinical and anatomical abnormalities [11,12]. Contrast agents are typically administered during imaging to improve the resolution of the area of interest. Iron oxide nanoparticles (IONPs) have been shown to be effective contrast agents in MRI, providing negative contrast in images [13]. By incorporating IONPs as the core in the preparation of multipurpose nanoparticles, they can be tracked in MRI [14,15]. Early and rapid detection of diseases is possible by using nanosystems for diagnostics. Nanoparticles such as gold, magnetic nanoparticles (MNPs), silica, polymeric micelles, and hydrogels have been widely used in the field of theranostics [10]. Silica is a very promising particle due to its biocompatibility and wide range of applications in medical systems [16,17,18]. The surface area for the adsorption of therapeutic molecules can be increased by introducing porosity into the silica structures. Mesoporous silica (MS) is a common porous silica structure with pore sizes between 2 and 50 nm. MSNs with a core of metal or metal oxide (MO)_n_, abbreviated as (MO)_n_@MSNs, have the combined properties of both elements [19]. MSNs with a core of IONPs have the potential to act as drug delivery vehicles that can be tracked using MRI [20].

Methotrexate (MTX) has been extensively studied and shown to be effective in the treatment of cancer, rheumatoid arthritis, and psoriasis. However, its therapeutic efficacy when administered via conventional drug delivery systems is hampered by its poor pharmacokinetics and limited safety margin [21]. To overcome these limitations, advanced nanocarriers and smart drug delivery systems have been developed and tested for tumor site-specific release of MTX [22]. Hydrogels have been shown to be an effective carrier for systemic and transdermal delivery of MTX [23]. Other platforms, including magneto-liposomes [24], magnetite–chitosan microspheres [25], hyperbranched polyglycerol-grafted MNPs [26], and arginine-capped MNPs [27], are examples of some reports that MTX was conjugated in their structure and have been used for controlled release and targeted delivery. An osteosarcoma-targeted codelivery system consisting of MTX-conjugated polyethylene glycol (PEG) and nanohydroxyapatite was found to be effective for chemotherapy [28]. MS-based materials, such as MCM-41 and aluminum-containing MCM-41, have been used for MTX drug delivery. However, they have not shown an adequate sustained release profile [29]. A transdermal system has been developed to deliver MTX via MSN, which successfully accumulates on the epidermis [30]. Smart delivery of MTX can be achieved by establishing an ester linkage that allows the drug to be released at the pH of the tumor site. Hydroxyl-modified magnetite nanoparticles and a polyhydroxyethylmethacrylate hydrogel have been embedded to fulfill this purpose in two of our previous investigations [31,32].

Glucose-conjugated nanoparticles or complexes are considered tumor-targeting agents [33,34]. The increase in glucose uptake by cancer cells is due to the overexpression of glucose transporters, in particular GLUT1 and GLUT3 [35]. 

Taking advantage of the development of MSN-based theranostic systems [10,36] and the novel application of MS-based materials as an antidote agent by Farjadian et al. [37,38,39], a novel synthesis of glucosamine-modified MS coated with MNP conjugated with MTX (MNP@MSN-MTX) is reported here. The system exhibited a pH-responsive pattern of MTX delivery and was evaluated for in vitro cytotoxicity, apoptosis induction, and cellular uptake in 4T1 cell lines. An in vivo mouse breast cancer model was developed, and MRI was used to follow the accumulation pathway of MNP@MSN-MTX.

## 2. Materials and Methods

### 2.1. Materials and Instruments

Materials required for this study were obtained from Sigma-Aldrich and Merck (Germany). MTX was purchased from EBEWE (Vienna, Austria). The products were analyzed by FT-IR spectroscopy using a Bruker VERTEX70 spectrometer (Ettlingen, Germany). X-ray diffraction (XRD) was performed using a 40 kV/MPD 3000. Analysis by vibrating sample magnetometer, VSM, was performed using a 7400 series (Meghnatis Danesh Pajouh/Iran) at 25 °C with a magnetic field of −5000 to 5000 Oe. Field emission scanning electron microscopy (FESEM) was performed using a MIRA3 TESCAN, and energy dispersive X-ray spectroscopy (EDX) was performed using a Tescan VEGA3. Elemental analysis was also carried out using a Costech Company’s CHNS-O elemental analyzer/ECS 4010 (Valencia/USA). Transmission electron microscopy (TEM) was carried out using a JEOL/JEM-1400 Plus. Dynamic light scattering (DLS) was used to determine hydrodynamic sizes using a Microtrac S3500 (York/USA). MTX concentration was determined using a UV–Vis spectrophotometer (CECIL CE7250). A multi-well plate reader/ELISA (Tecan Infinite M200 (Männedorf, Switzerland) was used to assess cytotoxicity, and data were analyzed using Excel 2013 and Curve Expert 1.4. A four-color FACSCalibur flow cytometer (BD Bioscience, NJ, USA) was used to assess the level of cellular uptake and the ability of the prepared nanoparticles to induce apoptosis in cancer cells. A Siemens 1.5 Tesla/Magnetom Avanto MRI machine (Erlangen, Germany) from Faghihi Hospital/Shiraz was used to image the mice. The MRI machine is equipped with a finger coil, and a specific pulse sequence was used to acquire the data.

### 2.2. Synthesis of Super-Paramagnetic Iron Oxide Nanoparticles (MNP)

Citric acid-coated nanomagnetite particles with diameters in sizes ranging between 10 and 12 nm were prepared according to the previously published procedure [40]. Briefly, FeCl_3_·6H_2_O (3.68 g) and FeCl_2_·4H_2_O (1.3 g) were admixed in 40 mL of degassed deionized water in a three-necked, round-bottom flask under a N_2_ atmosphere at 80 °C. The reaction mixture was sonicated with a 100-watt probe sonicator. After 30 min, an aqueous ammonia solution (25%, 20 mL) was injected into the medium, rapidly forming super-paramagnetic iron oxide. The mixture was sonicated for a further 30 min, then a trisodium citrate solution (4 mL, 0.5 g/mL) was injected into the flask, and the reaction was sonicated for more than 1 h. The product was centrifuged, washed three times with ethanol and finally with acetone, and dried overnight in an oven at 50 °C.

### 2.3. Synthesis of Glucosamine-Modified Mesoporous Silica-Coated MNP (MNP@MSN-Glu)

MNPs prepared by the previous method (0.3 g) were sonicated and dispersed in deionized water (500 mL) containing cetyltrimethylammonium bromide (5.4 mmol) and an aqueous ammonia solution (25%, 7.5 mL). After mixing for 15 min at 40 °C, an ethanolic solution (7.5 mL) containing TEOS (14.5 mmol) was added dropwise. The mixture was kept stirred at 80 °C for 48 h. The brownish mixture was centrifuged and washed with water and ethanol. It was lyophilized in a freeze-dryer. The product was calcined in an oven at 500 °C to remove CTAB and named MNP@MSN. The resulting product was then dispersed in ethanol (20 mL) and sonicated for 5 min, then 3-chloropropyltrimethoxysilane (16.4 mmol) was added, and the mixture was stirred at 40 °C for 24 h. The product was then separated by centrifugation, washed three times with ethanol and acetone, and dried by lyophilization. The product was named MNP@MSN-Cl.

To modify the surfaces with glucosamine, the product of this step containing 3-chloropropyl (0.05 g) was reacted with glucosamine hydrochloride (0.27 mmol) in the presence of trimethylamine (0.27 mmol) in a dimethylsulfoxide solution (2 mL) at 40 °C for 24 h. The product was then separated by centrifugation, washed with ethanol, and dried to give MNP@MSN-Glu.

### 2.4. Conjugation of MTX to MNP@MSN-Glu (MNP@MSN-MTX)

Conjugation of MTX to MNP@MSN-Glu was performed by Steglich esterification, similar to our previous report [31]. MTX (0.04 mmol) was activated by N, N′-dicyclohexylcarbodiimide/DCC (0.04 mmol), and a catalytic amount of DMAP (0.002 mmol) in 5 mL of dimethylsulfoxide for 1 h at room temperature. MNP@MSN-Glu (0.2 g) was then added and shaken for 24 h at 40 °C. The product was centrifuged and washed as before. In a desorption process, 100 µg/mL of MNP@MSN-MTX (2 mL) was prepared in a medium with pH = 1.5 and shaken in a thermal mixer for 48 h at 40 °C. The amount of MTX was determined by UV–Vis spectrophotometer at a wavelength of 306 nm wavelength. The encapsulation efficiency (EE%) and drug loading capacity (DLC%) were determined to be 10% and 33%, respectively.

### 2.5. Rhodamine Conjugation to MNP@MSN-Glu (MNP@MSN-Rhod)

Rhodamine B (Rhod) was attached to MNP@MSN-Glu through Steglich esterification, as in the first method, so that the fluorescent label could be added. Briefly, Rhod (0.062 mmol) was activated in a dimethylsulfoxide (5 mL) solution in the presence of DCC (0.062 mmol) and DMAP (0.008 mmol) for one hour at room temperature. MNP@MSN-Glu (130 mg) was then mixed with activated Rhod and stirred for 24 h at 40 °C. To purify the product (MNP@MSN-Rhod), the mixture was centrifuged, and the precipitate was separated, washed twice with ethanol and acetone, and dried in an oven at 50 °C.

### 2.6. MTX Release Profile

To evaluate the MTX release profile and stability to pH changes, microtubes containing 200 µg/mL MNP@MSN-MTX were dispersed in two media with pH values (5.5 and 7.4) for different periods (30 min, 1, 2, 4, 8, 12, 24, 48, and 72 h). The samples were shaken in a thermal mixer at 37 °C. After the defined time, the tubes were centrifuged, and the MTX absorbance was determined by UV–Vis spectroscopy.

### 2.7. Cell Lines and Cell Culture

Breast cancer cell line 4T1, a highly tumorigenic and invasive transplantable tumor cell line, was obtained from the National Cell Bank of Iran (NCBI, Pasteur Institute, Tehran, Iran). Cells were cultured in complete culture medium (RPMI 1640) containing 10% fetal bovine serum (FBS) and 1% penicillin/streptomycin (all from Gibco, USA) and incubated at 37 °C and five percent CO_2_ with 95% humidity. At 70–80% confluence, the culture medium was completely removed and washed with 1× sterile PBS (pH 7.2–7.4). Cells were then dissociated using a 0.25% trypsin–EDTA solution (Biosera, France), washed, and subjected to trypan blue dye exclusion staining for viability control and counting. The optimal number of cells was then prepared for injection and biological assays (MTT, apoptosis, and cellular uptake).

### 2.8. MTT Assay

The MTT (3-(4,5-dimethylthiazolyl-2)-2,5-diphenyltetrazolium bromide) cell proliferation assay, a commonly used method to measure cell proliferation and/or reduction in cell viability, was used to assess the cytotoxic activities of MTX, MNP@MSN-Glu, and MNP@MSN-MTX nanoparticles. In the first step, using a decreasing serial dilution of cells (1 × 10^6^ to 1 × 10^3^ cells per mL), the optimal cell number for the 4T1 cell line was 10 × 10^3^ cells. Accordingly, 10 × 10^3^ cells per well were seeded in 96-well microplates in RPMI 1640 complete medium. Three wells containing only medium were also included as blanks for absorbance measurements. Cells were treated with MNP@MSN-Glu (20–500 µg/mL), MNP@MSN-MTX (20–500 µg/mL), and MTX (2–50 µg/mL) in triplicate. After incubation for 48 h at 37 °C in a humidified CO_2_ incubator, the media were discarded, replaced with MTT solution (100 μL, 0.5 mg/mL), and incubated at 37 °C for 4 h. The medium was then aspirated to dissolve the formazan crystals, and DMSO (150 μL) was added to each well. The plate was incubated at 37° for 30 min in the dark C. The absorbance of all wells, including blanks, was measured spectrophotometrically at 492 nm. Data were analyzed using Excel 2013 and CurveExpert 1.4 software. The following formula was used to calculate the inhibitory effect of each compound. OD represents UV–Vis optical density.

$$Inhibition\left(\%\right)=100-\left(\frac{ODtest-ODblank}{ODnegative}\times 100\right)$$

The concentration with a 50% inhibitory effect was reported as the IC_50_ of each compound. Each experiment was repeated three times.

### 2.9. Apoptosis Assay

The PE Annexin V, Apoptosis Detection Kit I (B.D. Biosciences), was used to assess the ability of MNP@MSN-MTX nanoparticles to induce apoptosis in 4T1 cells. The day before treatment, 4T-1 cells were seeded with a density of 200 × 10^3^ in a complete culture medium into each well of a 24-well cell culture plate. After 24 h, the cells were treated with different concentrations of MTX, MNP@MSN-Glu, and MNP@MSN-MTX (0.5, 0.25, and 0.125 µg/mL). An untreated sample was also included as a negative control. After incubation period, the cells were detached, washed twice with cold 1× PBS (pH 7.2–7.4), suspended in 50 µL binding buffer, and transferred into flow cytometry tubes. Cells were stained with PE-conjugated Annexin V (2 µL) and 7-AAD (2 µL) solutions for the experimental tube and incubated for 15 min at RT under a dark cover. Unstained control cells were simply resuspended in a 50 µL binding buffer. After incubation for 15 min without washing, 300 µL of binding buffer was added to each tube and immediately analyzed on the flow cytometer. 

### 2.10. Drug Uptake by 4T1 Cells

The cellular uptake of particles by 4T1 cells was determined using rhodamine-conjugated particles (MNP@MSN-Rhod) and flow cytometry. Briefly, 4T1 cells were seeded at a density of 10 × 10^4^ in 1 mL of complete culture medium in flow cytometry tubes. The cells were then treated with MNP@MSN-Rhod (0.5, 2.5, 5, 10, 20, 50, and 100 ppm). One tube was left untreated and used as a negative control. The cells were incubated at 37 °C for 4 h, washed with 1× PBS, and immediately collected on a flow cytometer.

### 2.11. Establishment of an Orthotopic Mouse 4T1 Breast Tumor Model

Female 6–8-week-old BALB/c mice (NCBI, Pasteur Institute, Tehran, Iran) were used to establish a mammary tumor model. The animals were handled and housed according to the ethics and guidelines of Shiraz University of Medical Sciences (Ethical Code: IR.SUMS.REC.1395.S383). 

On the day of injection, 4T1 cells were detached and suspended at a density of 3 × 10^6^ in 500 µL of cold 1× PBS in an insulin syringe and kept on ice until injection. The hair around the nipples was shaved, and 4T1 cells were injected directly into the mammary glands. Tumors developed and were detectable after 20 days. Mass tumors were surgically removed from one mouse after 30 days and fixed in formalin to confirm tumor formation. Hematoxylin and eosin (H and E) staining was performed on different tumor tissue sections (4 μm). The growth of the tumors was monitored regularly, and when they reached 2 cm, they were subjected to intravenous (IV) injections of PBS (200 µL), MTX (200 µL, 833 µg/mL), and MNP@MSN-MTX (200 µL, 8333 µg/mL) every other day.

### 2.12. MRI Imaging 

MR images were acquired using a clinical 1.5 T scanner (MagnetOM Avanto, Siemens Healthcare, Germany). Fast spin-echo T2w images (Repetition Time (TR): 2800 ms and Time to Echo (TE): 95 ms) and gradient echo images (TR: 500 ms, TE: 26 ms, and flip angle: 20) were acquired in the coronal plane. The MRI images were obtained from three groups of mice with orthotopic tumors treated with PBS (control group), MTX, and MNP@MSN-MTX after 6 and 13 days of treatment. In the MNP@MSN-MTX-treated mice, an external magnet was placed on the chest to facilitate the accumulation of MNP@MSN-MTX in the chest.

## 3. Results

### 3.1. Synthesis and Characterization of MNP@MSN-MTX

**Figure 1:**
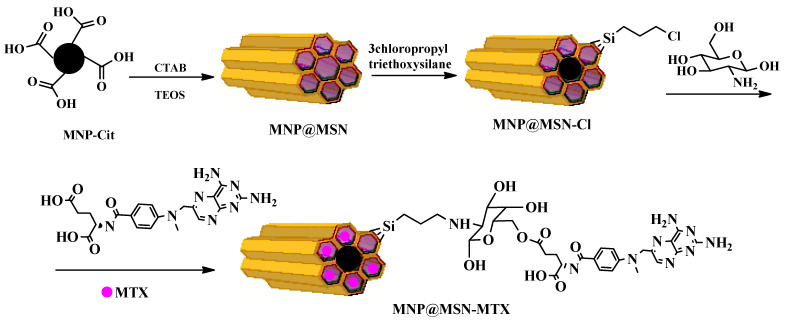
Schematic illustration of synthetic steps regarding the formation of MNP@MSN, MNP@MSN-Glu, MNP@MSN-MTX.

**Figure 2:**
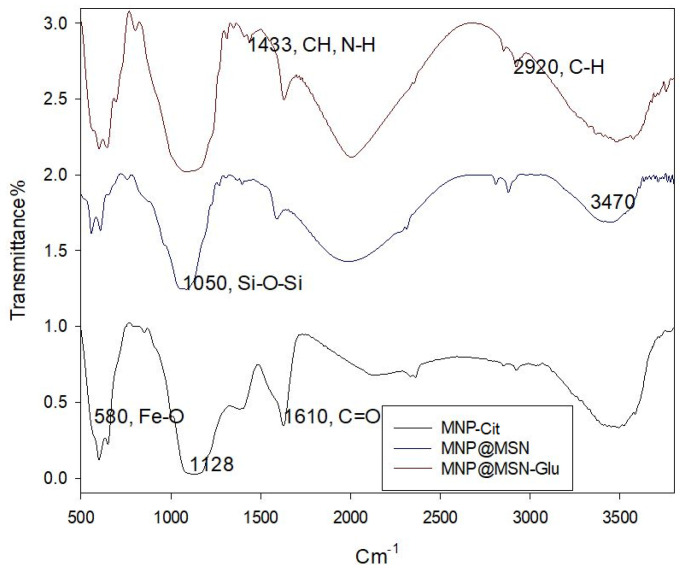
FT-IR spectra of MNPCit, MNP@MSN, and MNP@MSNGlu.

The sodium citrate-stabilized magnetic nanoparticles (MNP-Cit) with sizes between 8 and 10 nm were synthesized in a single-step approach by co-precipitation of iron ions in an alkaline solution [40]. The synthesis strategy for preparing MNP@MSN-MTX nanoparticles is illustrated in Figure 1. MNP-Cit nanoparticles were coated with MSNs in the presence of TEOS as a silane precursor and CTAB as a templating agent to produce biocompatible nanoparticles with a high surface area. After the calcination process to remove CTAB, the porous structure MNP@MSN was functionalized with chloropropyl through a reaction with 3-chloropropyl trimethoxysilane to obtain MNP@MSN-Cl. Furthermore, to introduce glucosamine to the surfaces, MNP@MSN-Cl was reacted with glucosamine hydrochloride to form MNP@MSN-Glu. Finally, MTX was conjugated to the glucose hydroxyl groups to produce MNP@MSN-MTX (Figure 2). 

To detect the structural changes via different functional groups’ formation after each modification step, FT-IR spectroscopy was utilized. MNP-Cit’s FT-IR spectra showed the stretching vibration of Fe-O at 580 cm^−1^ and the asymmetric stretching vibration band of C=O, which belongs to the citric groups, at 1610 cm^−1^, while MS coating (MNP@MSN) resulted in the appearance of a broad band at 1050–1200 cm^−1^ belonging to a Si-O-Si asymmetric stretching vibration. In the spectrum of MNP@MSN-Glu, the bending vibration of the C-H groups of glucosamine appears at 1433 cm^−1^ and the asymmetric vibration of C-H at 2920 cm^−1^.

The successful structural changes are demonstrated by the results of the CHN elemental analysis as shown in Table 1.

**Figure 3:**
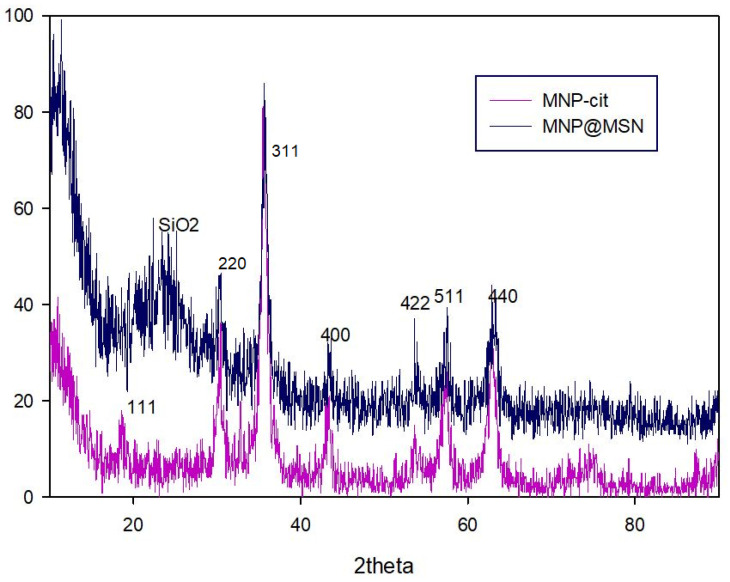
XRD patterns of MNP-Cit and MNP@MSN; *hkl* indices are shown in the MNP@MSN pattern.

XRD analysis is an effective method for evaluating the crystal structure of compounds, chemical changes, and crystallinity of the grain structure. The XRD patterns of the particles, MNP-Cit and MNP@MSN, were defined (Figure 3). The diffraction rings of Fe_3_O_4_ can be attributed to (111), (220), (311), (400), (422), (511), and (440), indicating that the synthesized MNPs have a high degree of conformity with the single crystalline phase of Fe_3_O_4_ as well as cubic spinel (JCPDS #894319, 19-0629). Consequently, after the preparation of MNP@MSN, a broad peak appeared in the 2θ = 20 corresponding to amorphous silica (SiO_2_). The modification processes did not significantly change the intensity of the MNP-Cit peaks, indicating that the crystal forms of Fe_3_O_4_ did not change.

**Figure 4:**
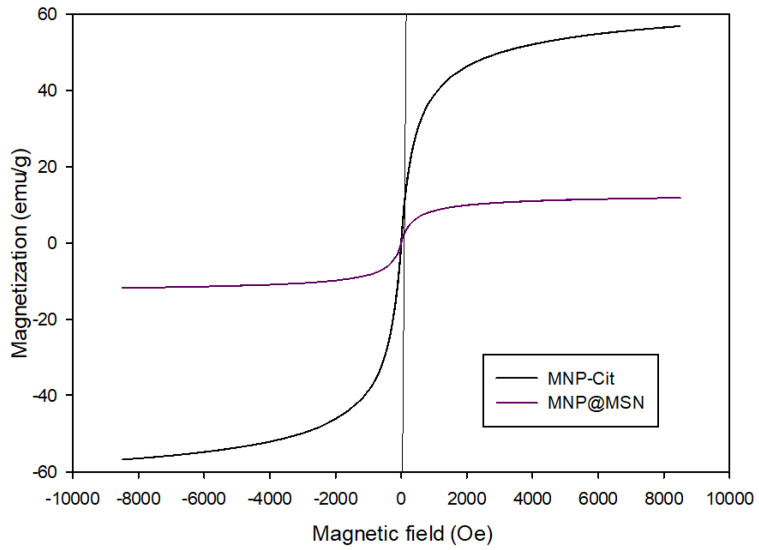
VSM graph of MNPCit and MNP@MSN.

A VSM was used to study the magnetic properties of the nanoparticles. From the diagram, it was found that the saturation magnetization (Ms) for MNP-Cit is 55 emu/g and decreased to 12 emu/g after the creation of MNP@MSN (Figure 4). This indicates that the particles have good magnetic properties and could be used to deliver MTX or other drugs using a magnetic field. Keshavarz et al. reported that suitable saturation magnetization for biomedical purposes (i.e., hyperthermia and drug delivery) is in the range of 8–19 emu/g [41].

Textural analysis was carried out with the N_2_ adsorption/desorption technique, and the parameters for samples (i.e., MNP@MSN and MNP@MSN-Glu) were derived from BET and BJH models. The N_2_ adsorption and desorption plots with the BET surface area and BJH adsorption cumulative pore volume are presented in SI-Figures S1 and S2. The derived parameters are listed in Table 2.

**Figure 5:**
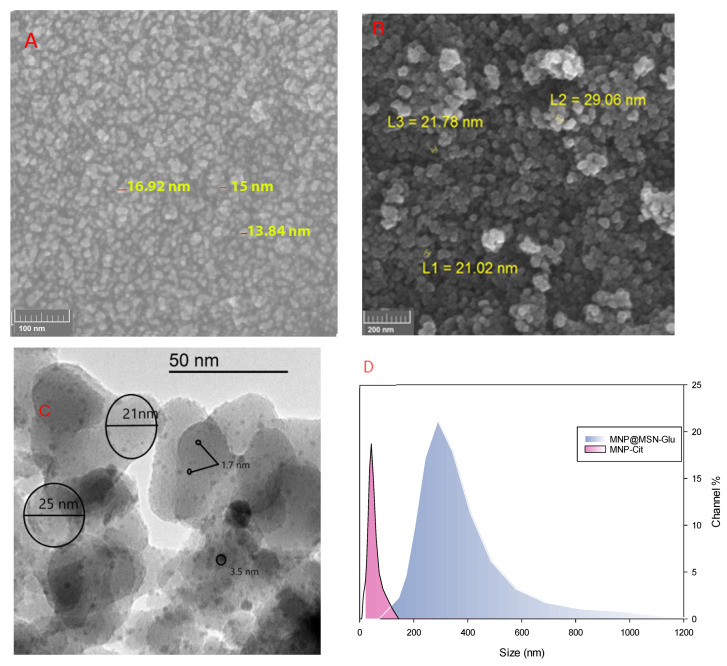
Size assessment: (**A**) SEM image of MNP-Cit, (**B**) SEM image of MNP@MSN, (**C**) TEM image of MNP@MSN-Glu, and (**D**) DLS analysis of MNP-Cit and MNP@MSN-Glu.

Size analysis is essential to determine the functional capability of nanoparticles in biological systems. Here, the sizes of MNP and MNP@MSN-Glu were determined with several techniques, including FESEM, TEM, and DLS. The size of MNP-Cit was around 15 nm (Figure 5A), and the particles became larger when modified with silica precursors. The FESEM image (Figure 5B) shows that MNP@MSN has a size of 20 nm, as demonstrated by FESEM. DLS analysis shows that MNP-Cit has a fine distribution around particle sizes of 44 nm (Figure 5A). The TEM image (Figure 5C) shows that MNP@MSN-Glu particles have a fine distribution of MNP, and MSN structures are well-formed. The particle sizes of the spheres are around 20 nm. MNP-Cit particles are dispersed like raisins on a cake within the MSN structure with sizes less than 10 nm. DLS analysis shows MNP@MSN-Glu particles with sizes around 322 nm (Figure 5D).

### 3.2. MTX Conjugation and Release Profile

All the evidence gathered from the structural analysis of MNP@MSN-Glu shows that these particles could provide a platform for magnetic field-stimulated drug delivery. The presence of glucosamine on the surface of this structure is considered a safe site for the conjugation of therapeutic agents. By this means, the anticancer drug MTX was conjugated through an esterification reaction among the available glucosamine hydroxyl group and the MTX carboxylic acid group. The reaction was conducted in the presence of DCC as an activating agent and DMAP as a catalyst. The amount of MTX on the surface of MNP@MSN-MTX was determined to be 10% of the total weight by UV–Vis analysis in desorption processes.

**Figure 6:**
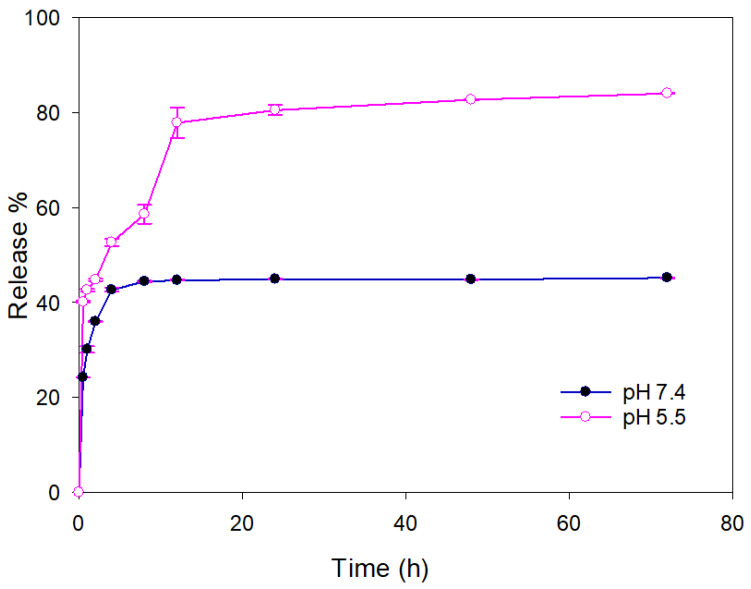
MTX release percent from MNP@MSN-MTX.

Control over drug release is crucial for nano-engineered particles with therapeutic goals. In this study, MTX release from MNP@MSN-MTX was studied at two pH values of 7.4 and 5.5, which are characteristics of physiological and tumoral microenvironments, respectively (Figure 6). MTX showed a maximum release of 40% in medium with a pH of 7.4 over a 72 h period, while more than 80% release was observed in medium with a pH of 5.5 over the same period.

### 3.3. Cellular Uptake 

**Figure 7:**
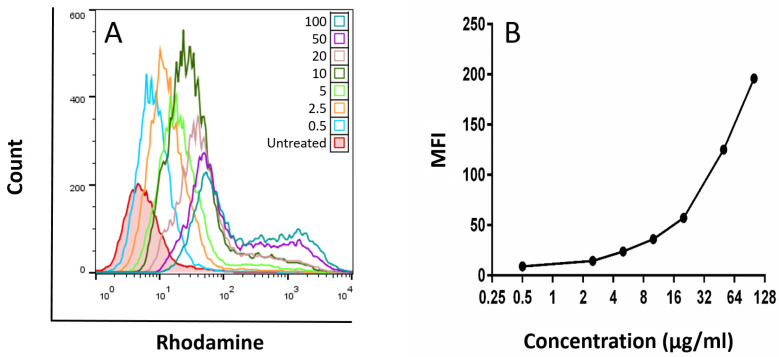
Flow cytometric analysis of MNP@MSN-Rhod cellular uptake by the 4T1 cell line over a 4 h period. (**A**) A histogram of flow cytometric analysis represents the intensity of the fluorochrome (Rhodamine). The number on the y-axis shows the percentage of cells with the same fluorescence intensity, and the x-axis is the fluorescence intensity. (**B**) Representation of the mean fluorescence intensity (MFI) (y-axis) in 4T1 cells with increasing concentration of MNP@MSN-Rhod.

As indicated in Figure 7A,B, with the increase in the concentration of MNP@MSN-Rhod from 0 (untreated cells: red) to 100 µg/mL (blue-green), the mean fluorescence intensity (MFI) of Rhodamine in the cells was also increased. These results showed effective uptake of MNP@MSN-Rhod by 4T1 cells at low to high concentrations. 

For 4 h, 4T1 cells were treated with different concentrations of MNP@MSN-Rhod, and the MFI of Rhodamine was used as an index of drug accumulation in the cell compared to untreated cells (negative control; red).

### 3.4. Cytotoxic Effect of MNP@MSN-Glu on 4T1 Cell Line

**Figure 8:**
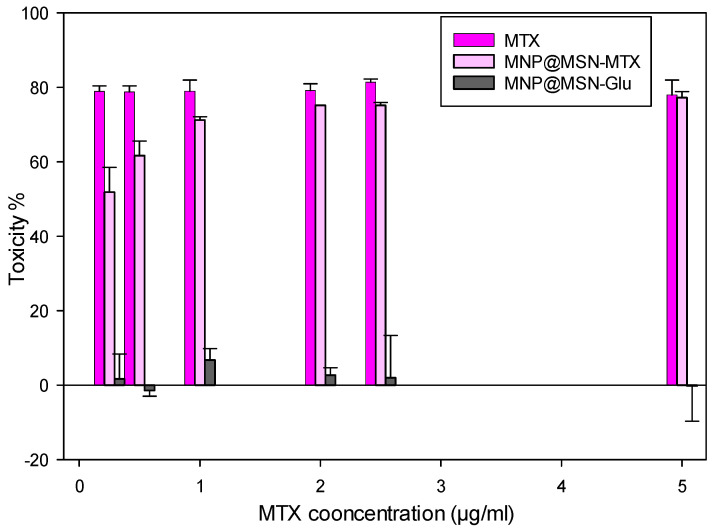
In vitro toxicity assessment of MTX, MNP@MSNMTX, and MNP@MSNGlu on 4T1 breast cancer cell line at 24 h.

The cytotoxic effects of MTX, MNP@MSN-MTX, and MNP@MSN-Glu were evaluated on 4T1 cells using an MTT assay. The results showed that MTX at different concentrations from 0.25 to 5 µg/mL had similar inhibitory effects, averaging 78.54 ± 2.78 (77–81%). In contrast, MNP@MSN-MTX showed dose-dependent inhibitory effects with an IC_50_ of 0.28 ± 0.10. MNP@MSN-Glu was also evaluated, and the results showed no inhibitory or cytotoxic effect in the range of 0.25–5 µg/mL, and the cells showed increased proliferation for MNP@MTX-Glu particles (Figure 8). 

**Figure 9:**
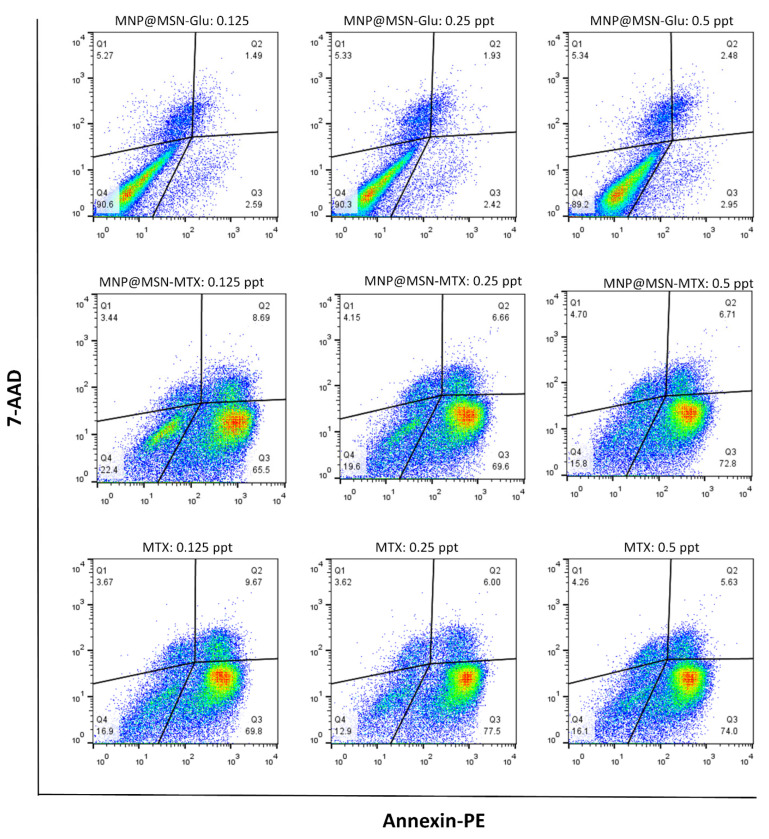
Apoptotic effect of MNP, MNP-MTX, and MTX on 4T1 cells after 48 h of treatment.

We then tested the apoptotic effect of the particles on the 4T1 cell line (Figure 9). While MTX and MNP-MTX significantly induced apoptosis on 4T1 cells at different concentrations in a dose-independent manner, no significant apoptotic effect was observed in the case of MNP@MSN-Glu alone (less than 10%). The results also confirmed that the conjugation of MTX to MNP@MSN-Glu did not affect the activity of MTX in inducing apoptosis.

For 48 h, 4T1 cells were treated with MNP@MSN-Glu, MNP@MSN-MTX, and MTX and then stained with Annexin V and 7-AAD. Cells that were negative for both Annexin V and 7-AAD were considered healthy (Q4), and cells that were negative for 7-AAD but positive for Annexin V were defined as early apoptotic cells (Q3). 7-AAD+ Annexin V+ cells (Q2) were considered cells in the late apoptotic phase, and those stained with 7-AAD only were considered necrotic cells (Q1). The results are shown in Figure 9.

### 3.5. In Vivo Experiments and MRI Analysis

**Figure 10:**
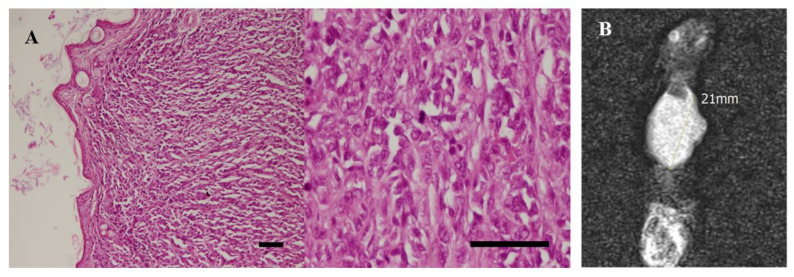
(**A**) Histopathological image of breast cancer tumor with scale bar (scale bar is equivalent to 100 micrometers), and (**B**) pre-treatment T2w MR image of the tumor initially shows a 21 mm lobulated hypersignal mass.

In vivo tumor analysis showed that a tumor mass was detected 20 days after the 4T1 injection. The histopathological evaluation also confirmed the presence of tumor cells in the detected mass (Figure 10A), indicating successful tumor development. MRI analysis also indicated that the breast cancer tumor had been successfully induced, and the images showed that the tumor had an effect size of 21 mm (Figure 10B).

**Figure 11:**
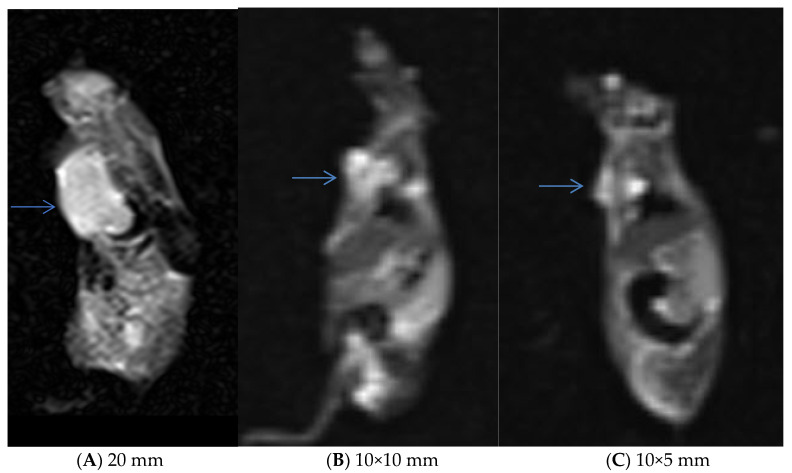
The second MRI performed 6 days after the initiation of the treatment. Stable size of the mass in the control, blue arrows show tumors (**A**) and a significant decrease in the size of the lesions in the treated tumors (**B**,**C**) (“(**B**)” was the case treated with MTX, and “(**C**)” was treated with MNP@MSN-MTX in the presence of external magnet).

Further MRI assessments were performed after treatment in tumor-induced models. The mice were divided into three groups: control, MTX, and MNP@MSN-MTX, and were injected every other day with PBS, MTX, and MNP@MSN-MTX, respectively. In this phase of this study, the second MRI scan was taken six days after the start of treatment. Significant reductions in the tumor size were observed in the MTX- and MNP@MSN-MTX-treated groups (Figure 11).

**Figure 12:**
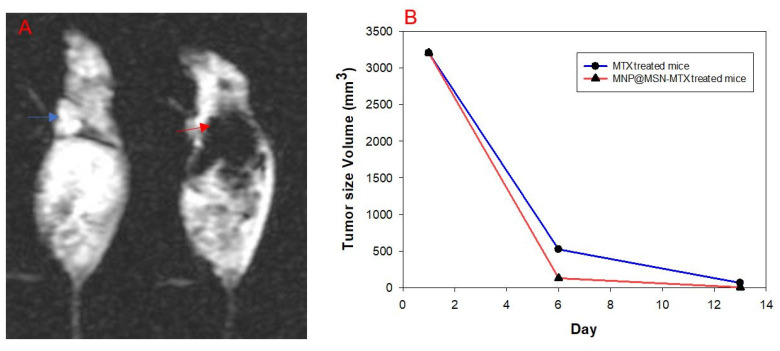
(**A**) Images with gradient echo technique acquired from the treated groups demonstrate a lower signal of the tumor (red arrow), which was treated with the use of MNP@MSN-MTX, indicating the susceptibility effect of local accumulation of the particles in the tumor via external magnet, compared with the MTX-treated case (blue arrow). (**B**) Diagram showing changes in tumor size over the time interval of MRI monitoring.

The ability of MNP@MSN-MTX to contrast was carefully assessed using gradient echo in the MRI performed on day 6 of treatment (Figure 12). Negative contrast was observed in the breast tumors in the MNP@MSN-MTX-treated group compared to the MTX-treated group.

**Figure 13:**
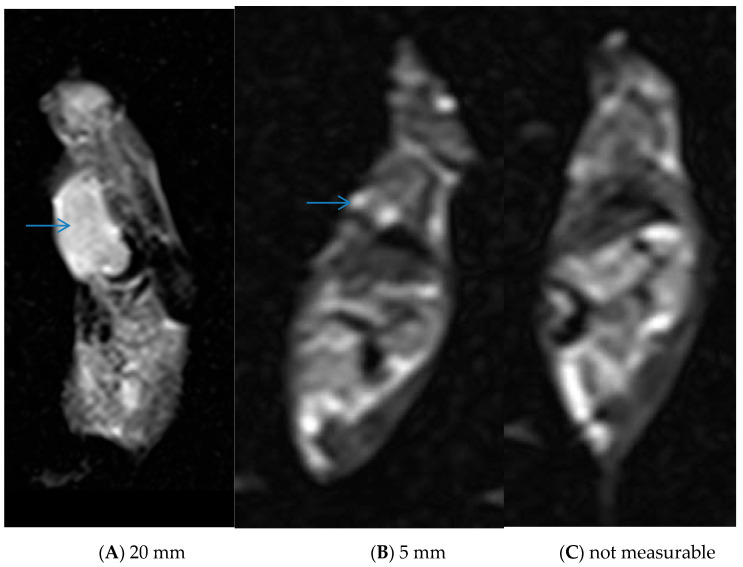
The third MRI, performed 13 days after treatment, demonstrates (**A**) stable size of the tumor in the control and further decrease in the tumor size in the treated group (**B**,**C**). Only a small residual tissue is identifiable (blue arrow) after 13 days ((**A**,**B**); blue arrow).

Continuing treatment, the third MRI was performed 13 days after the injection (Figure 13). Careful evaluation showed that tumor size had decreased to 5 mm in the MTX-treated group and was undetectable in the MNP@MSN-MTX group. A graph showing the changes in tumor size over the time interval of MRI monitoring is shown in Figure 12B.

## 4. Discussion

A refined synthetic process has led to the development of a biocompatible drug delivery carrier that can be easily tracked using MRI. The process involved the creation of super-paramagnetic iron oxide particles through a co-precipitation reaction, followed by a citrate coating to act as a stabilizer and protective agent. The particles were then treated with a silane coupling agent for functionalization. A controlled size synthesis was performed by dispersing the particles in an aqueous ammonium medium and adding an ethanolic solution of TEOS to form MNP@MSN. The resulting material, known as a “raisin-cake” structure, was further functionalized by reaction with 3-chloropropyltrimethoxysilane and glucosamine to create MNP@MSN-Glu. Esters were then formed to attach an anticancer drug, MTX, to the structure. Each step was characterized using FT-IR spectroscopy, XRD, VSM, and CHN analysis. The resulting MNP@MSN-Glu showed promising properties for MRI and drug delivery, with a reduced magnetization but sufficient sensitivity to magnetic fields. The controlled-size synthesis of MSN silica played a critical role in the development of injectable nanoformulations, with the MNP@MSN particles having a hydrodynamic diameter of approximately 313 nm and a “raisin-cake” structure. “Raisin cake” is a new term first introduced for the first time in this manuscript by Farjadian et al. The fine distribution of MNPs in a mesoporous spherical structure of MSN is the advantage of such a structure. The structure retains the properties of MNPs, such as magnetization and contrast ability in MRI. At the same time, it has no toxicity, which is the main problem with MNP carriers [42]. The endocytosis pathway of magnetic mesoporous silica (M-MSNP) might be different depending on its shape. In a study by Shao et al., a rod-like and a sphere-like M-MSNP library were prepared. Particles of both shapes had good biocompatibility; however, the rod-like particles showed more accumulation in HepG2 tumors in in vivo models [43]. This finding demonstrates the importance of shapes in the endocytosis pathway.

This research holds promise for the smart delivery of MTX, reducing the side effects associated with free drugs. It was found that 1.1 mmol/g of methotrexate (MTX) was chemically bound to the structure of the nanoparticles. The release behavior of the system indicated that it is responsive to changes in pH, with a higher release rate observed in an acidic medium at a pH of 5.5 compared to a physiological medium at a pH of 7.4. The biological effects of the MTX-conjugated nanoparticles (MNP@MSN-Glu) were evaluated in the 4T1 cell line. The cytotoxicity assay showed that the MNP@MSN-MTX particles exhibited significant cytotoxicity even at the lowest concentration of MTX. The IC_50_ value was determined to be 0.28 µg/mL in a dose-dependent manner, while the cytotoxicity of MTX remained constant over all concentrations. In addition, the nanoparticles with glucose amino groups (MNPs-glucose amins) showed no inhibitory or cytotoxic effects within the tested range. Interestingly, MNP@MSN-Glu was found to induce cell proliferation, which may be attributed to the presence of glucose as a nutrient for the cells.

Further investigations, including cellular uptake and apoptosis assays, were conducted to elucidate the mechanisms of action of MNP@MSN-Glu on cancer cells. The cellular uptake assay showed efficient uptake of fluorescence-labeled particles (MNP@MSN-Glu) by cells, while increasing the concentration did not hinder the uptake process. The results of the apoptosis assay indicated that the conjugation of MTX to the nanoparticles did not significantly affect the apoptotic induction of MTX.

Breast cancer tumors were induced in mice by injection of 4T1 cells. Successful tumor formation was confirmed by pathological examination and MRI analysis. After tumor formation, the mice were divided into three groups: control, MTX, and MNP@MSN-MTX. Treatment was started, and the efficacy of the treatment was monitored by measuring the tumor size using MRI. After six days of treatment, the tumor size was reduced by half, and on day 13, the MNP@MSN-MTX-treated mouse showed complete tumor regression, while the MTX-treated mouse still had a 5 mm tumor. There were some challenges in evaluating the treatment in vivo. The survival rate of the mice in the MTX and MNP@MSN-MTX groups was 75% in the first 6 days, but after 13 days, the survival rates dropped to 50% and 25%, respectively. An unexpected observation was made in the PBS-treated group where the mice became blind. This study paves the way for future projects focusing on more detailed in vivo delivery of such particles. Overall, MTX treatment is challenging because patients are always at risk of overdosing, which can be fatal [37]. In this scenario, MTX delivery holds great promise. MSN particles are particularly noteworthy, as they can be extensively modified and tailored for imaging purposes by incorporating gold, quantum dots, and magnetic nanoparticles at their core [10,36]. 

## 5. Conclusions

In this project, a new theranostic system using MNP-modified MSN was introduced. An innovative approach was to modify the structure with glucosamine, resulting in a high abundance of hydroxyl groups on the surface. An esterification reaction with hydroxyl groups was used to conjugate an anticancer drug, MTX. The characterization of the product was confirmed with FT-IR and CHN analyses. Magnetization is crucial for the application of MNP particles, and VSM analysis revealed that MNP had a high magnetization of about 55 emu/g, which decreased to 12 emu/g after modification with MSN. Despite the decrease, the particles remained suitable for magnetic field drug delivery. XRD analysis confirmed the formation of Fe_3_O_4_ structures in both MNP and MNP@MSN particles, with an appropriate size range of 40–50 nm for MNP@MSN. MTX demonstrated excellent pH-sensitive release from MNP@MSN-MTX in a simulated tumor medium compared to the physiological pH. Both MTX and MNP@MSN-MTX exhibited high toxicity towards 4T1 cells, while MNP@MSN-Glu showed biocompatibility. Cellular uptake was observed over a range of concentrations, and apoptotic induction was observed for both free and conjugated MTX. The efficacy of 4T1 cells to induce breast cancer in BALB/C mice was confirmed, and MRI was utilized as a diagnostic tool for the treatment of mice with breast cancer using MTX and MNP@MSN-MTX. Continuous administration of MNP@MSN-MTX resulted in tumor regression within 13 days, and the anticancer efficacy with contrast ability in MRI was confirmed with all the results obtained.

## Figures and Tables

**Table 1:** Elemental analysis of MNP@MSN-Glu and MNP@MSN-MSTX.

Sample	Nitrogen%	Carbon%	Hydrogen%
MNP@MSN-Glu	0.43	15.96	4.63
MNP@MSN-MTX	1.45	15.06	3.43

**Table 2:** Results of textural analysis of MNP@MSN and MNP@MSN-Glu.

Sample	Surface Area (m^2^/g) (BET Calculation)	Pore Volume (cm^3^/g) Single Point ads/des	Pore Width (nm) (BJH Calculation)
MNP@MSN	644	0.58	3.6
MNP@MSN-Glu	310	0.22	2.9

Adsorption/desorption (ads/des).

## Data Availability

All data are presenetd in the text or avaible as Supporting Information.

## Supplementary Materials

The following supporting information can be downloaded at: https://www.mdpi.com/article/10.3390/pharmaceutics15102491/s1, Figure S1: Porosity characterization of MNP@MSN-Glu, N2 Adsorption/desorption (a), BET surface area plot (b), BJH adsorption cumulative pore volume (c); Figure S2: Porosity characterization of MNP@MSN-Glu, N2 Adsorption/desorption (a), BET surface area plot (b), BJH adsorption cumulative pore volume (c).
