# Supplementary material for: Glucosamine-Modified Mesoporous Silica-Coated Magnetic Nanoparticles: A “Raisin-Cake”-like Structure as an Efficient Theranostic Platform for Targeted Methotrexate Delivery
Source: Pharmaceutics. 2023 Oct 19;15(10):2491. doi: 10.3390/pharmaceutics15102491 (PMC10610088; doi:10.3390/pharmaceutics15102491)
Supplement: Supplementary file 1 [file pharmaceutics-15-02491-s001.zip › pharmaceutics-2586372-supplementary.pdf]

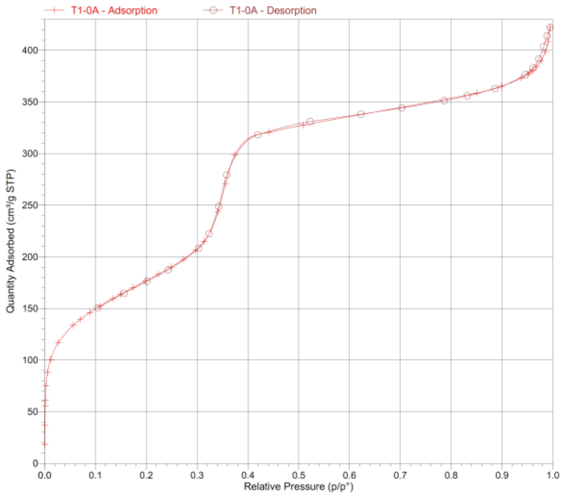

(a)

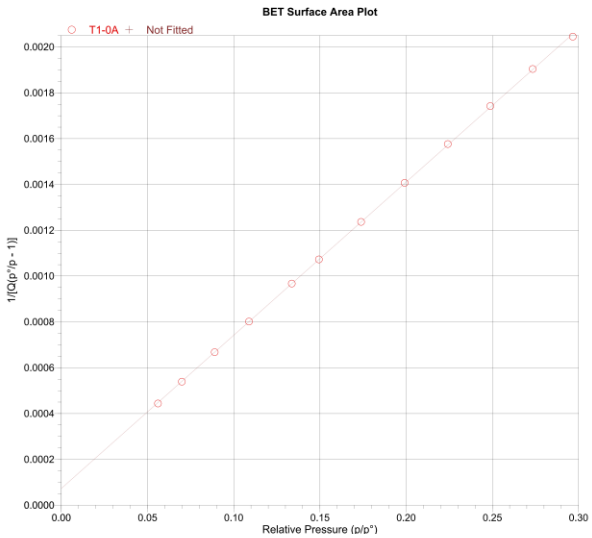

(b)

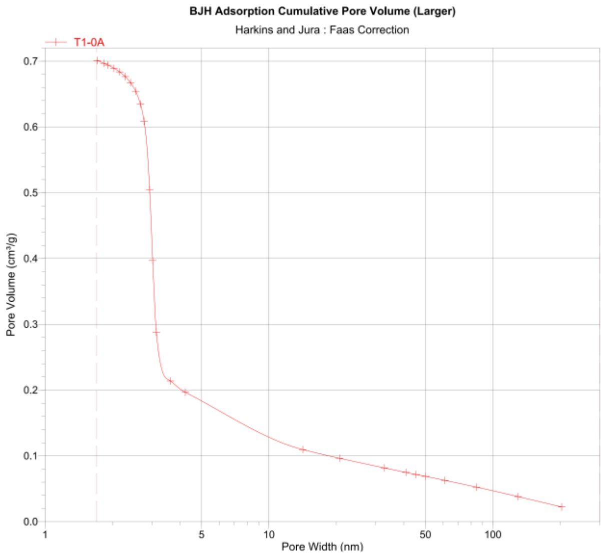

(c)

Figure S1- Porosity characterization of MNP@MSN-Glu, N_2 Adsorption/desorption (a), BET surface area plot (b), BJH adsorption cumulative pore volume (c).

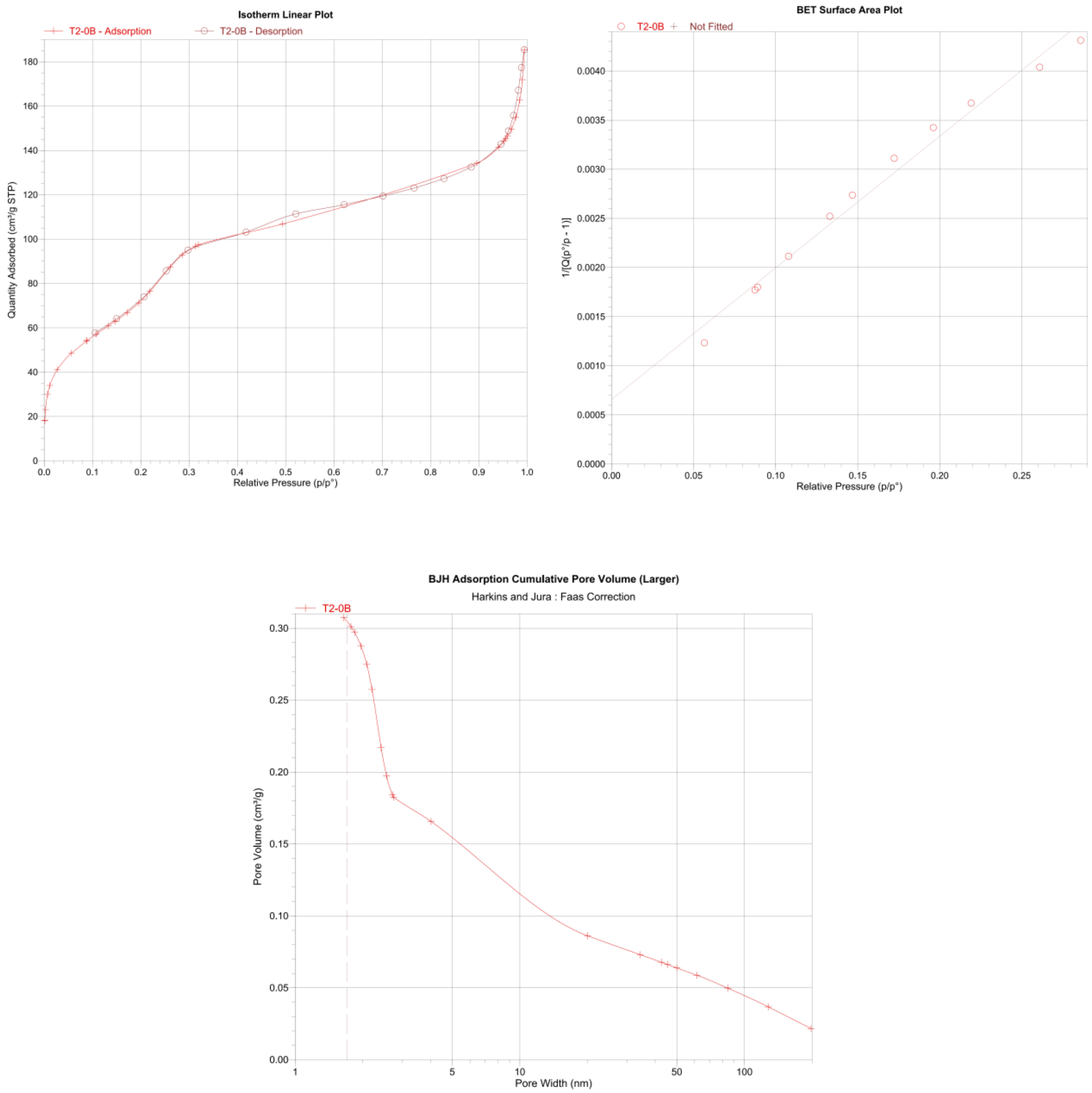

Figure S2- Porosity characterization of MNP@MSN-Glu, N_2 Adsorption/desorption (a), BET surface area plot (b), BJH adsorption cumulative pore volume (c).
